# Comparative analysis of EZH2, p16 and p53 expression in uterine carcinosarcomas

**DOI:** 10.3389/pore.2023.1611547

**Published:** 2023-12-11

**Authors:** Evelin Makk, Noémi Bohonyi, Angéla Oszter, Klára Éles, Tamás Tornóczky, Arnold Tóth, Endre Kálmán, Krisztina Kovács

**Affiliations:** ^1^ Department of Pathology, University of Pécs Medical School, Pécs, Hungary; ^2^ Department of Obstretrics and Gynaecology, University of Pécs Medical School, Pécs, Hungary; ^3^ Department of Medical Imaging, University of Pécs Medical School, Pécs, Hungary

**Keywords:** uterine cancer, carcinosarcoma, EZH2, p16, p53

## Abstract

**Introduction:** The role of p16 and p53 immunohistochemistry in the diagnosis of rare and aggressive uterine carcinosarcoma (UCS) has been well established. However, enhancer of zeste homolog 2 (EZH2), a histone methyltransferase and a member of the polycomb group family is a relatively new biomarker, with limited published data on its significance in this tumor type. The goal of this study was to examine EZH2 expression in UCS and its components, in correlation with morphological features, and p16 and p53 staining patterns.

**Methods:** Twenty-eight UCSs were included in the study. EZH2, p16 and p53 immunoreactivity were assessed independently by two pathologists in both tumor components (epithelial and mesenchymal). EZH2 and p16 immunostains were scored semiquantitatively: based on the percentage and intensity of tumor cell staining a binary staining index (“high- or low-expressing”) was calculated. The p53 staining pattern was evaluated as wild-type or aberrant (diffuse nuclear, null, or cytoplasmic expression). Statistical tests were used to evaluate the correlation between staining patterns for all three markers and the different tumor components and histotypes.

**Results:** High EZH2 and p16 expression and aberrant p53 patterns were present in 89.3% 78.6% and 85.7% of the epithelial component and in 78.6%, 62.5% and 82.1% of the mesenchymal component, respectively. Differences among these expression rates were not found to be significant (*p* > 0.05). Regarding the epithelial component, aberrant p53 pattern was found to be significantly (*p* = 0.0474) more frequent in the serous (100%) than in endometrioid (66.6%) histotypes. Within the mesenchymal component, p53 null expression pattern occurred significantly (*p* = 0.0257) more frequently in heterologous sarcoma components (71.4%) compared to the homologous histotype (18.8%).

**Conclusion:** In conclusion, EZH2, p16 and p53 seem to play a universal role in the pathogenesis of UCS; however, a distinctive pattern of p53 expression appears to exist between the serous and endometrioid carcinoma components and also between the homologous and heterologous sarcoma components.

## Introduction

Uterine carcinosarcoma (UCS), or uterine malignant mixed Müllerian tumor (MMMT) is a very aggressive and rare neoplasm of the female genital tract, comprising less than 5% of endometrial malignancies [1].

This tumor has a biphasic morphology, containing both malignant epithelial (carcinomatous) and malignant mesenchymal (sarcomatous) components [2]. In most cases, it consists of a single carcinoma and a single homologous sarcoma histologic subtype. The former is usually serous carcinoma, followed by less common endometrioid, clear cell, undifferentiated, and mixed histotypes. The mesenchymal component is most often a homologous high grade sarcoma, and less often it consists of a heterologous rhabdomyosarcoma (RMS), chondrosarcoma, osteosarcoma, or liposarcoma [3, 4].

The majority of studies support the “metaplastic monoclonal or conversion theory,” whereby UCS develops from the metaplastic transformation of a single neoplastic cell type [5]. As part of the process, epithelial-mesenchymal transition (EMT) allows a polarized epithelial cell to transmogrify into a mesenchymal cell phenotype, giving the ability for it to migrate away from its original epithelial layer [6]. The EMT theory is supported by high epithelial to mesenchymal transition gene signature scores and is likely due to epigenetic alterations at microRNA promoters and histone gene mutations and amplifications [7]. However, a small percentage of UCS seem to represent real collision tumors, since they are molecularly biclonal and most likely develop from two independent cell populations [8].

Carcinosarcomas exhibit a significantly poorer prognosis compared to other high-grade endometrial carcinomas such as grade 3 endometrioid carcinoma, serous carcinoma and clear cell carcinoma [9].

Polycomb group proteins are a group of important epigenetic regulators. Enhancer of zeste homologue 2 (EZH2), a histone lysin methyltransferase and a catalytic component of polycomb repressive complex 2 is involved in cell proliferation, cell differentiation and tumorigenesis by silencing the transcription of several tumor suppressor genes (including p21, p16 and p27) [10–13]. Accordingly, numerous studies have highlighted the role of EZH2 in cancer development and progression. Overexpression of EZH2 protein has been shown in various malignant tumors, including carcinomas of the breast [14], lung [15], stomach [16], colon [17], pancreatobiliary tract [18], liver [19], thyroid gland [20], prostate [21], and bladder [22]. EZH2 has been also studied in most common gynecologic malignancies such as cervical [23, 24], endometrial [25, 26] and ovarian cancer [27, 28].

In recent years, EZH2 expression has also been discovered in certain sarcomas, including Ewing sarcoma [29], RMS [30, 31], synovial sarcoma [32], osteosarcoma [33], and chondrosarcoma [34].

Strong evidence demonstrated that EZH2 could promote EMT [35, 36], therefore, we postulated that aberrant EZH2 overexpression may also be invoved in the pathogenesis of UCS.

Nevertheless, only limited data are available regarding EZH2 expression and its clinicopathological correlations in UCS. EZH2 positivity in UCS was previously only reported in one effusion cytology specimen [37].

p53 is a frequently used immunohistochemical marker in the diagnostic work-up of endometrial carcinomas, and based on The Cancer Genome Atlas database over 90% of UCS harbor TP53 mutation [38]. Similarly, the p16-RB pathway has also been previously implicated in the pathogenesis of UCS [39].

In this study, our goal was to investigate the potential role of EZH2 along with p16 and p53 biomarkers in the diagnosis and histogenesis of UCS components and their histotypes.

## Materials and methods

### Subjects

The study was approved by our institutional ethical committee (number of permission: KK/644-1/2020). Consecutive cases of UCS diagnosed from 2012 to 2019 were retrieved from the archives of the Department of Pathology, University of Pécs, Hungary.

Formalin-fixed and paraffin embedded tissue samples were collected from hysterectomy and biopsy (curettage) specimens. The original hematoxylin and eosin–stained slides from each case were reviewed, and representative blocks containing both malignant epithelial and mesenchymal components were selected for immunohistochemistry (IHC). The epithelial component was subclassified according to the current WHO classification [40] as serous, endometrioid, clear cell, undifferentiated and mixed histotypes, while the mesenchymal component was classified as homologous or heterologous type.

### Immunohistochemical analysis of EZH2, p16 and p53 expression

For IHC, 4 µm thick sections were cut from the formalin-fixed paraffin-embedded tissue specimens. Immunostaining for EZH2 (mouse monoclonal, clone 6A10, prediluted, Newcastle Upon Tyne, United Kingdom), for p16 (mouse monoclonal, clone E6H4; Ventana Medical System Inc., Tucson, AZ), and for p53 (rabbit monoclonal, clone SP5, prediluted; Thermo Scientific, United States) were performed with proper positive and negative controls using Leica Bond Max autostainer (Leica Biosystems, Bannockburn, IL) and Leica Bond Polymer Refine Detection Kit (Upon Tyne, United Kingdom). Immunoreactivity was evaluated in both the epithelial and mesenchymal components independently by two board-certified pathologists with over 15 years of professional experience (K.K., A.O.).

EZH2 and p16 expression were scored semiquantitatively according to the percentage of tumor cell nuclear staining: 1+: <10% of tumor cell nuclei, 2+: between 10% and 50% of tumor cell nuclei, 3+: >50% of tumor cell nuclei [24, 41–44], and staining intensity (0: no staining, 1+: weak, 2+: moderate, 3+: strong). As in the previous studies, a staining index was calculated as the product of staining percentage and intensity on a scale of 0–9 [45, 46]. The tumors were categorized as high-expressing (staining index >4) or low-expressing (staining index ≤4).

P53 immunoreactivity interpretation was conducted according to the recommendations by the International Society for Gynecological Pathologists [47]. The p53 staining pattern was evaluated as wild-type or aberrant (latter can be further classified as diffuse nuclear, null, or cytoplasmic expression) [48]. In more details, wild-type (normal) pattern is met when a scattered nuclear staining is present with no or weak cytoplasmic staining; Aberrant/diffuse nuclear pattern is met when 80% strong and diffuse nuclear staining is present (with or without any cytoplasmic staining), Aberrant/null pattern is met when a complete absence of nuclear staining in all cells is present without cytoplasmic staining; Aberrant/cytoplasmic pattern is met when a moderate to strong cytoplasmic staining is present in the absence of diffuse nuclear expression.

### Statistical evaluation

For EZH2 and p16, the median staining index scores (low or high expression) of the two experts were calculated in both components. Regarding staining percentage and staining intensity, the ratings were summed up for further analysis. Discordant p53 pattern ratings were reevaluated and the final result was decided in consensus. The EZH2 and p16 expression indices and p53 staining patterns were compared between the epithelial and mesenchymal components, as well as between the serous and endometrioid carcinoma components, and between the homologous and heterologous sarcoma components. Mixed and undifferentiated epithelial histotypes were excluded from histotype comparisons due to the low case numbers. Depending on the sample size, Chi square or Fisher exact tests were used to compare categorical values (staining index and p53 staining pattern) while Mann-Whitney test was used to compare ordinal values (staining percentage and staining intensity). Concordance between epithelial and mesenchymal components were evaluated with kappa test. All statistical tests were run in Medcalc [49]. A *p*-value of <0.05 was considered statistically significant [50].

## Results

This study included 28 women with uterine carcinosarcoma, with a median age of 70.5 years (range 53–85 years). Formalin-fixed and paraffin embedded tissue samples were available from 22 hysterectomy and 6 biopsy (curettage) specimens. Histologically, the epithelial component comprised of 14 (50%) serous, 9 (32.1%) endometrioid, 2 (7.1%) undifferentiated, and 3 mixed (serous and endometrioid) carcinomas (10.7%). The mesenchymal component contained heterologous elements in 7 cases (25%), and homologous elements in 21 samples (75%). Among the heterologous sarcoma components, 4 chondrosarcoma and 3 RMS occurred. The most common homologous components were endometrial stromal sarcoma (*n* = 14), followed by 3 leiomyosarcomas, 3 undifferentiated sarcomas, and 1 myxoid fibrosarcoma.

The immunoreactivity measures of EZH2 and p16 in the epithelial and mesenchymal components are shown in Table 1. High EZH2 expression was slightly more common in the epithelial (89.3%) than in the mesenchymal (78.6%) component. Similarly, p16 expression was slightly higher in the epithelial (78.6%) compared to the mesenchymal (62.5%) components. Based on Fisher’s exact test, p16 and EZH2 expressions between the epithelial and mesenchymal components were not statistically different (for EZH2 *p* = 0.468; for p16 *p* = 0.248). Based on Mann-Whitney tests, marker staining percentages and intensities were also not statistically different between the epithelial and mesenchymal components (*p* = 0.074 and *p* = 0.076 for staining percentage of EZH2 and p16, respectively; *p* = 0.11 and *p* = 0.059 for staining intensity of EZH2 and p16, respectively).

**TABLE 1:** Immunoreactivity of EZH2 and p16 in UCSs based on the two independent experts’ ratings (medians for staining index, sums for staining percentage and nuclear intensity).

	EZH2		p16	
	Epithelial component	Mesenchymal component	Epithelial component	Mesenchymal component
Staining index	n (%)			
Low^a^	3 (10.7)	6 (21.4)	6 (21.4)	10.5 (37.5)
High^b^	25 (89.3)	22 (78.6)	22 (78.6)	17.5 (62.5)
	average^c^(SD)			
Staining percentage	5.68 (0.72)	5.18 (1.15)	4.96 (1.68)	4.32 (1.87)
Nuclear Intensity	5.64 (0.95)	5.18 (1.36)	5.21 (1.5)	4.46 (1.86)

^a^ Staining index of ≤ 4.

^b^ Staining index of > 4.

^c^ Value range = 0-6.

**FIGURE 1:**
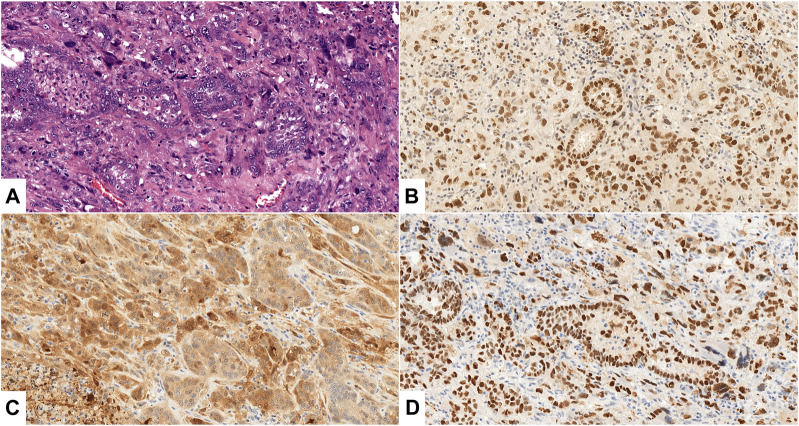
**(A–D)**: Uterine carcinosarcoma, Case No. 26., ×200 magnification. **(A)** HE staining shows malignant epithelial (serous carcinoma) and mesenchymal (homologous undifferentiated sarcoma) components. **(B, C)** Diffuse, strong positive (3+) expression of EZH2 **(B)**, p16 **(C)** in both components; **(D)**: >80% strong and diffuse p53 nuclear staining in both components.

Figure 1 shows representative cases of diffuse and strong immunoreactivity with EZH2 and p16 markers and diffuse nuclear p53 pattern.

The immunoreactivity measures of EZH2 and p16, in the different carcinoma components are shown in Table 2. Both serous and endometrioid histotypes showed prominent staining with all markers. High expression of EZH2 was almost always present (89% for both endometrioid and serous carcinomas). High p16 expression was also very common (88% of endometroid, 79% of serous cases). There were no statistical differences in the EZH2 and p16 staining scores between the serous and endometrioid histotypes (Fisher exact test *p* values for staining index of EZH2 and p16 between the histotypes were 0.74 and 0.96 respectively. Mann-Whitney *p* values for staining percentages of EZH2 and p16 between the two carcinoma histotypes were 0.35 and 0.15, respectively. Mann-Whitney *p* values for EZH2 and p16 staining intensity between the two carcinoma histotypes were 0.37 and 0.26, respectively.)

**TABLE 2:** Immunoreactivity of EZH2 and p16 in UCS epithelial component serous and endometrioid histotypes based on the two independent experts’ ratings (medians for staining index, sums for staining percentage and nuclear intensity).

	EZH2				p16			
	Endometrioid	Serous	Mixed	Undiff.	Endometrioid	Serous	Mixed	Undiff.
Staining index	n (%)							
Low^a^	1 (11)	1.5 (10.7)	0.5 (16.7)	0 (0)	2 (22)	3 (21)	1 (33.3)	0 (0)
High^b^	8 (89)	12.5 (89.3)	2.5 (83.3)	2 (100)	7 (88)	11 (79)	2 (66.7)	2 (100)
	average^c^ (SD)							
Staining percentage	5.77 (0.66)	5.57 (0.85)	5.66 (0.57)	6 (0)	4.88 (1.45)	4.93 (1.9)	4.66 (2.3)	6 (0)
Nuclear Intensity	5.44 (1.33)	5.71 (0.82)	5.66 (0.57)	6 (0)	5.11 (1.76)	5.28 (1.32)	4.66 (2.3)	6 (0)

^a^ Staining index of ≤4.

^b^ Staining index of >4.

^c^ Value range = 0–6.

The immunoreactivity measures of EZH2 and p16 in homologous and heterologous sarcoma components are shown in Table 3. Both sarcoma types showed similarly common high EZH2 expression (79% of the homologous elements, and 78.6% of the heterologous elements) and p16 expression (60% of the homologous elements and 72% of the heterologous elements). These markers showed no statistical differences (Fisher exact test *p* values for staining index of EZH2 and p16 between the two types were 1 and 0.66, respectively. Mann-Whitney *p* values for staining percentage of EZH2 and p16 between the two types were 0.37 and 0.4, respectively. Mann-Whitney *p* values for staining intensity of EZH2 and p16 between the two types were 0.1 and 0.48, respectively.)

**TABLE 3:** Immunoreactivity of EZH2 and p16 in UCS mesenchymal component homologous and heterologous types based on the two independent experts’ ratings (medians for staining index, sums for staining percentage and nuclear intensity).

	EZH2		p16	
	Homologous	Heterologous	Homologous	Heterologous
Staining index	n (%)			
Low^a^	4.5 (21)	1.5 (21.4)	8.5 (40)	2 (28)
High^b^	16.5 (79)	5.5 (78.6)	12.5 (60)	5 (72)
	average^c^ (SD)			
Staining percentage	5.14 (1.23)	5.28 (0.95)	4.23 (1.92)	4.57 (1.81)
Nuclear Intensity	5.38 (1.28)	4.57 (1.51)	4.42 (1.98)	4.57 (1.51)

^a^ Staining index of ≤4.

^b^ Staining index of >4.

^c^ Value range = 0–6.

P53 staining patterns are shown in Table 4. Aberrant p53 immunostaining was seen in 85.7% of the epithelial and 82.1% of the mesenchymal components. These values were statistically equal (Fischer test *p* = 1). P53 cytoplasmic staining was observed only focally in 3/28 cases, but none of the tumors showed aberrant cytoplasmic expression pattern. Within the epithelial components, aberrant p53 expression was found in 66.6% of the endometrioid and 100% of the serous carcinomas, which was statistically significant (Fisher test *p* = 0.0474). Regarding the mesenchymal components, homologous sarcomas showed aberrant p53 patterns in 76.2% of cases, while all heterologous sarcomas (100%) were p53 aberrant (no statistical difference, Fisher test *p* = 0.29). Aberrant null p53 pattern was observed in 71.4% of the heterologous sarcomas, whereas only 18.8% of the homologous sarcomas displayed this staining pattern (statistically significant difference, Fisher test *p* = 0.0257). Aberrant diffuse nuclear p53 expression was seen in 81.2% of the homologous sarcoma histotypes. No other significant differences were identified in p53 patterns among the UCS components and their different histotypes.

**TABLE 4:** p53 staining patterns in UCS components and component types.

		Epithelial component					Mesenchymal component		
Expression		Endometrioid	Serous	Mixed	Undifferentiated	All	Homologous	Heterologous	All
Wild-type^a^		3/9 (33.3%)	0/14 (0%)	0/3 (0%)	1/2 (50%)	4/28 (14.3%)	5/21 (23.8%)	0/7 (0%)	5/28 (17.9%)
Aberrant^b^		6/9 (66.6%)	14/14 (100%)	3/3 (100%)	1/2 (50%)	24/28 (85.7%)	16/21 (76.2%)	7/7 (100%)	23/28 (82.1%)
Pattern	Diffuse nuclear	5/6 (83.3%)	11/14 (78.6%)	2/3 (66.7%)	0/1 (0%)	18/24 (75%)	13/16 (81.2%)	2/7 (28.6%)	15/23 (65.2%)
	Null	1/6 (16.7%)	3/14 (21.4%)	1/3 (33.3%)	1/1 (100%)	6/24 (25%)	3/16 (18.8%)	5/7 (71.4%)	8/23 (34.8%)
	Cytoplasmic	0/6 (0%)	0*/14 (0%)	0/3 (0%)	0/1 (0%)	0*/24 (0%)	0/16 (0%)	0/7 (0%)	0/23 (0%)

^a^ Wild-type (normal) - scattered nuclear staining).

^b^ Aberrant/Diffuse nuclear pattern: 80% strong and diffuse nuclear staining with ot without cytoplasmic staining, Aberrant/null pattern: complete absence of nuclear staining in all cells, Aberrant/cytoplasmic pattern: moderate to strong cytoplasmic staining in the absence of diffuse nuclear expression. *One serous case and two endometrioid cases presented weak to moderate cytoplasmic staining but also at least 80% strong and diffuse nuclear staining, therefore were attributed diffuse nuclear pattern (see Figure 2).

**FIGURE 2:**
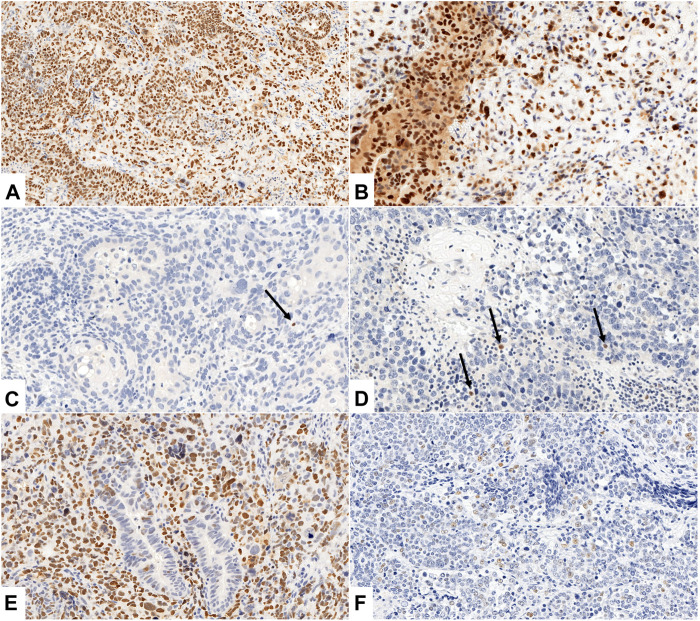
**(A–F)** Different patterns of p53 expression. **(A, B)** Aberrant, p53 diffuse nuclear pattern with weak **(A)** and weak to moderate **(B)** cytoplasmic staining in epithelial and mesenchymal components of UCS (**(A)**: Case No. 26., ×200 magnification, **(B)**: Case No. 5., ×400 magnification). **(C, D)** Aberrant, p53 null pattern with complete absence of nuclear staining in all cells of both components (**(C)**: Case No. 11., ×200 magnification, **(D)**: Case No. 21., ×200 magnification). Arrows indicate internal positive controls (lymphocytes). **(E, F)** Wild type p53 IHC pattern with scattered nuclear staining in epithelial **(E)** and mesenchymal **(F)** component, **(E)**: aberrant, p53 diffuse nuclear pattern in mesenchymal component (**(E)**: Case No. 19., ×200 magnification, **(F)**: Case No. 22., ×400 magnification).

Figure 2 illustrates different patterns of p53 expression.

P53 was concordant between epithelial and mesenchymal components in 82.14% (Cohen’s k: 0.34, fair agreement).

## Discussion

Our results showed that EZH2 and p16 are similarly highly expressed, while p53 immunostaining is aberrant in the majority of uterine carcinosarcomas. Pattern differences were found between histotypes.

Previous studies have described various immunohistochemical profiles of carcinosarcomas.

EZH2 positivity in UCS was previously reported in a single effusion cytology specimen in one study [37], which investigated the utility of EZH2 as a single immunomarker in the diagnosis of metastatic carcinoma in effusion samples. A total of 108 pleural, peritoneal, and pericardial effusions/washings diagnosed as unequivocally reactive (*n* = 41) and metastatic carcinoma (*n* = 67) by cytomorphology over 18 months were reviewed. Among the metastatic carcinomas, 54 cases were adenocarcinoma and the remaining cases were squamous cell carcinoma (*n* = 1), carcinosarcoma (*n* = 1), and carcinoma of undefined histological subtypes (*n* = 11). Only one carcinosarcoma cytology specimen was included in this study that was a positive case.

The role of p16 and p53 immunohistochemistry as diagnostic and prognostic markers in UCS has been evaluated by a handful of prior studies. Engelsen et al. demonstrated that pathologic expression of p53 and p16 in endometrial curettings identifies high-risk endometrial carcinoma patients with poor prognosis [51]. Buza and Tavassoli reported p16 overexpression in the carcinomatous and sarcomatous components of uterine MMMTs, and observed that p16 immunohistochemical reaction was significantly more intense and diffuse than p53 immunostaining in both components. The concordance rates for p16 and p53 immunoreactivity for the two components within the same tumor were 83% and 90% of cases, respectively [39]. Xiaowei et al. examined the possible utility of p16, p53, and PAX8 IHC in the diagnosis of carcinosarcomas, and revealed almost equal staining in both components for p16 and p53. P16 staining showed almost equally high expression in the epithelial (74%) and mesenchymal components (71%), and p53 expression also was similar in the epithelial (48%) and mesenchymal (44%) components. High-expression of PAX8 was more common in the epithelial (73%) than in the mesenchymal (13%) components [46].

Compared to PAX8, EZH2 in our study shows not only a slightly higher expression rate in the epithelial component but is similarly highly expressed in the mesenchymal component as well.

Increased expression of EZH2 has been described in most common gynecologic malignancies such as cervical [23, 24], endometrial [25, 26] and ovarian cancer [27, 28]. Prognostic significance of EZH2 expression status has been also reported in patients with these tumours, because high expression of EZH2 was associated with tumor aggressiveness [52–54]. Therefore, EZH2 has been raised as a potential target for tumor therapy, and both preclinical and clinical studies on EZH2 inhibitors are intensively pursued [36, 55–58]. Regarding EZH2 as potential therapeutic target in UCS, no data exists in the literature.

Several further immunohistochemical markers have been studied as potential markers/adjuncts in the diagnosis of UCS as well, including p27, c-KIT, COX-2, EGFR, C-ErbB-2, the oncogene AKT [39, 59–63]. Recently, HER2 expression was also demonstrated in carcinosarcoma, as a potential therapeutic marker. Rates of HER2 overexpression or amplification were reported to be ranging from 6% to 25%. Intratumoural heterogeneity of HER2 expression/amplification and lower HER2 expression in the sarcoma component compared to the carcinoma component was also shown [62, 64, 65].

Regarding endometrial cancers in general, it is well-documented that the staining patterns of p53 and p16 are different between endometrial serous and endometrioid carcinomas [66–68]. The primary emphasis in most studies examining p53 in endometrial carcinomas is placed on the correlation between p53 overexpression and serous histology [69]. Mutations in TP53 are observable in intraepithelial carcinomas and are believed to occur at an early stage in the development of uterine serous carcinoma [70]. In a subset of endometrioid carcinomas, p53 overexpression has been documented, predominantly in tumors categorized as FIGO grade 3 endometrioid carcinomas [71]. In line with a 2018 recommendation, tumors exhibiting a double negative profile (p16-negative/p53-wild-type) are indicative of being primarily endometrioid, whereas tumors demonstrating a double positive profile (p53 aberrant/mutation-type and diffuse strong p16 positive) are more likely associated with serous histology [66]. This is consistent with the findings of the present study as we found significant more (*p* = 0.0474) p53 aberrant type serous than endometrioid subtype of the epithelial UCS components. The different p16 and p53 staining patterns between endometrial cancer types and UCS related epithelial component histotypes raise the possibility of their different pathomechanisms. In non-UCS related endometrial cancers, overexpression of EZH2 is well established [26, 72]. Gu et al [72] collected a total of 104 samples from patients with the diagnosis of endometrial cancer and analyzed the expression of EZH2 by immunohistochemical staining. The results showed that the positive expression rate of EZH2 was 68.27%, which was significantly higher than that in the adjacent tissue (*p* < 0.05). Krill et al [26] analysed 87 tissue specimens from sixty patients with both early and advanced stage endometrioid endometrial adenocarcinoma and 27 matched-normal tissue specimens. Their results showed that EZH2 mRNA (*p* < .0001) and protein expression (*p* < .0001) in tumor specimens were significantly higher than in the matched-normal tissue. In primary tumors, EZH2 protein expression was associated with lympho-vascular space invasion (*p* = .044), and EZH2 mRNA expression was associated with age (*p* = .037). However, these studies did not include specific cohorts of carcinosarcoma.

The pathogenesis of UCS is still debated. Authors largely agree that EMT seems to play role in its development [3, 5, 6, 73–75]. The EZH2-PRC2 complex regulates several target genes. EZH2 can directly bind to important tumor oncogenes and initiate signaling pathways for EMT events. Furthermore, EZH2 can induce EMT. Ding et al. demonstrated that the EZH2 inhibitor GSK343 suppresses the progression of cervical cancer cells by inhibiting EMT. Their data also established that treatment with GSK343 leads to a suppression of EMT in xenograft tumours in nude mice. The observed EZH2–EMT-associated phenotypes and their underlying mechanisms have important implications for cervical cancer development and severity, which suggests that targeting this pathway through specific inhibitors would result in general epigenetic reprogramming [55]. Our data support the possible role of EZH2 in the pathogenesis of UCS along with the EMT theory.

The carcinomatous and sarcomatous components showed 85% concordance of p53 protein overexpression and 96% concordance of TP53 gene mutation, which points to a monoclonal origin of both components. P16 overexpression occurs in about 60% of UCS simultaneously with TP53 mutations. The concordance of p16 expression between the carcinomatous and sarcomatous components was approximately 85% in different series [39, 76].

P53 IHC is widely used as a surrogate for TP53 mutation testing in diagnostic gynecologic pathology [77]. P53 IHC is a reliable diagnostic adjunct for histotyping and molecular subtyping of endometrial carcinomas [48, 66, 78]. Another use of p53 IHC is triaging gynecological sarcomas for molecular testing based on the assumption that TP53-mutated gynecological sarcomas do not harbor cancer driving translocations [77]. Kunc et al. have observed high frequency of aberrant p53 IHC expression in extrauterine carcinosarcoma and high concordance between the carcinomatous and sarcomatous components [79]. Liu et al. reported p53 overexpression in 63% of UCS, with 47% and 77% overexpression in the homologous and heterologous tumors, respectively [80]. It has been recently noted that 46% of the epithelial and 53% of the mesenchymal components showed overexpression of p53, displaying a strong similarity of these tumor components [81]. In general, p53 diffuse positive cases indicate possible nonsynonymous missense mutations. In contrast, stopgain, indel or splicing mutations seem to result p53 null positive phenotype [82–84].

We observed a high frequency of aberrant p53 IHC expression in UCS with a fair concordance (82.14%, Cohen’s k = 0.34) between the epithelial and mesenchymal components that is in line with previous reports [79–81, 85].

Cherniack et al. identified multiple somatic mutations and copy number alterations in UCSs that offer expanded therapeutic options including potential use of PARP, EZH2, cell cycle and PI3K pathway inhibitor [7]. Jones et al. in their study performed a complete exome analysis of 22 UCS and verified genetic alterations in chromatin remodelling genes. Overall, they identified 777 somatic mutations in 702 genes. Mutations in EZH2 were observed in 12% of the mutant tags [86].

The most frequently mutated genes in carcinosarcomas are TP53, PTEN, PIK3CA, RB1, PPP2R1A, FBXW7, KRAS and ARID1A [7]. In TCGA, over 90% of carcinosarcomas harbor a TP53 mutation [7]. Some carcinosarcomas share mutational profiles with the endometrioid lineage, e.g., PTEN mutation, indicating that, like serous carcinoma, an alternative route of carcinogenesis is via a low-grade endometrioid carcinoma and its precursors [87]. A comprehensive analysis of the genomic and proteomic profiles of 57 UCSs has unveiled disruptions in canonical pathways, notably the PI3K pathway. More than 75% of cases demonstrated mutations in FBXW7, loss of RB1, or amplification of CCNE1, suggesting dysregulation in cell cycle control [7].

Mutations in TP53 are frequently observed across various tumors, underscoring the pivotal involvement of the FBXW7, p53, and PI3K pathways in UCS. Among these pathways, FBXW7 stands out as a crucial driver in the development of this particular cancer [88]. Conclusive genetic evidence obtained through lineage tracing studies indicates that UCS originates from endometrial epithelial cells undergoing an epithelial-mesenchymal transition. This transition gives rise to a highly invasive phenotype, with FBXW7 identified as the specific driver in this process [89]. In accordance with the conversion theory, UCS are believed to originate in a monoclonal fashion, with carcinomatous subclones having the potential for metaplastic differentiation and subsequent transformation into sarcomatous cells [7]. Support for this theory stems from the concurrent expression of cytokeratins and epithelial membrane antigens in both carcinomatous and sarcomatous cells. Additionally, there is consistency in TP53 and KRAS mutations, identical patterns of X chromosome inactivation, and comparable loss of heterozygosity observed between the carcinomatous and sarcomatous components [7].

The main limitation of our study is that detailed molecular pathological exploration of EZH2, p53 and p16 and their interactions were not performed, which may be the focus of future research. In addition, statistical power may be impacted by the low case number, however, this is largely explained by the rarity of UCS. The applied cutoff values for defining EZH2 and p16 as low or high expressing are somewhat arbitrary and may affect case distributions. However, to date no standardized scoring has been adopted for these markers. So, instead of adjusting cutoff values for our study, we aimed to adhere to previous publications to achieve better reproducibility and comparability [24, 41–44]. Strengths of the study include that both components of UCS as well as their subtypes were analyzed.

In conclusion, EZH2, p16 and p53 seem to play a universal role in the pathogenesis of UCSs. However, a distinctive pattern of p53 expression appears to exist between the serous and endometrioid types of the epithelial component (aberrant vs. wild type) and also between the homologous and heterologous types of the mesenchymal component (diffuse nuclear aberrant vs. null aberrant). These pattern disparities may indicate unique genetical features and differentiation pathways warranting further molecular pathological studies.

## Data Availability

The raw data supporting the conclusion of this article will be made available by the authors, without undue reservation.

## Abbreviations

MMMT: Malignant mixed Müllerian tumor; UCS: Uterine carcinosarcoma; EMT: Epithelial-mesenchymal transition; EZH2: Enhancer of zeste homologue 2; RMS: Rhabdomyosarcoma; IHC: Immunohistochemistry; ec: epithelial component; mc: mesenchymal component.

## References

[1] Loda M Mucci LA Mittelstadt ML Hemelrijck MV Cotter MB. Pathology and epidemiology of cancer. Cham: Springer International Publishing (2016). 10.1007/978-3-319-35153-7

[2] Silverberg SG Major FJ Blessing JA Fetter B Askin FB Liao SY Carcinosarcoma (malignant mixed mesodermal tumor) of the uterus: a gynecologic oncology group pathologic study of 203 cases. Int J Gynecol Pathol (1990) 9(1):1–19. 10.1097/00004347-199001000-00001 2152890

[3] Artioli G Wabersich J Ludwig K Gardiman MP Borgato L Garbin F. Rare uterine cancer: carcinosarcomas: review from histology to treatment. Crit Rev Oncol Hematol (2015) 94(1):98–104. 10.1016/j.critrevonc.2014.10.013 25468677

[4] Leskela S Pérez-Mies B Rosa-Rosa JM Cristobal E Biscuola M Palacios-Berraquero ML Molecular basis of tumor heterogeneity in endometrial carcinosarcoma. Cancers (Basel) (2019) 11:964. 10.3390/cancers11070964 31324031 PMC6678708

[5] Kernochan LE Garcia RL. Carcinosarcomas (malignant mixed müllerian tumor) of the uterus: advances in elucidation of biologic and clinical characteristics. J Natl Compr Cancer Netw (2009) 7(5):550–6. 10.6004/jnccn.2009.0037 19460280

[6] Kalluri R Weinberg RA. The basics of epithelial-mesenchymal transition. J Clin Invest (2009) 119(6):1420–8. 10.1172/JCI39104 19487818 PMC2689101

[7] Cherniack AD Shen H Walter V Stewart C Murray BA Bowlby R Integrated molecular characterization of uterine carcinosarcoma. Cancer Cell (2017) 31(3):411–23. 10.1016/j.ccell.2017.02.010 28292439 PMC5599133

[8] George E Lillemoe TJ Twiggs LB Perrone T. Malignant mixed müllerian tumor versus high-grade endometrial carcinoma and aggressive variants of endometrial carcinoma: a comparative analysis of survival. Int J Gynecol Pathol (1995) 14(1):39–44. 10.1097/00004347-199501000-00007 7883424

[9] Zhang C Hu W Jia N Li Q Hua K Tao X Uterine carcinosarcoma and high-risk endometrial carcinomas: a clinicopathological comparison. Int J Gynecol Cancer (2015) 25(4):629–36. 10.1097/IGC.0000000000000350 25633654

[10] Hock H. A complex Polycomb issue: the two faces of EZH2 in cancer. Genes Dev (2012) 26(8):751–5. 10.1101/gad.191163.112 22508723 PMC3337450

[11] Xie Z Zhong C Shen J Jia Y Duan S. LINC00963: a potential cancer diagnostic and therapeutic target. Biomed Pharmacother (2022) 150:113019. 10.1016/J.BIOPHA.2022.113019 35462329

[12] Sasaki M Nakanuma Y. Cellular senescence in biliary pathology. Special emphasis on expression of a polycomb group protein EZH2 and a senescent marker p16INK4a in bile ductular tumors and lesions. Histol Histopathol (2015) 30(3):267–75. 10.14670/HH-30.267 25289642

[13] Said J. Biomarker discovery in urogenital cancer. Biomarkers (2005) 10(1):83–6. 10.1080/13547500500215050 16298916

[14] Pourakbar S Pluard TJ Aaccurso AD Farassati F. Ezh2, a novel target in detection and therapy of breast cancer. Onco Targets Ther (2017) 10:2685–7. 10.2147/OTT.S138777 28579806 PMC5449122

[15] Findeis-Hosey JJ Huang J Li F Yang Q McMahon LA Xu H. High-grade neuroendocrine carcinomas of the lung highly express enhancer of zeste homolog 2, but carcinoids do not. Hum Pathol (2011) 42(6):867–72. 10.1016/j.humpath.2010.09.019 21292308

[16] Choi JH Song YS Yoon JS Song KW Lee YY. Enhancer of zeste homolog 2 expression is associated with tumor cell proliferation and metastasis in gastric cancer. APMIS (2010) 118(3):196–202. 10.1111/j.1600-0463.2009.02579.x 20132185

[17] Fluge O Gravdal K Carlsen E Vonen B Kjellevold K Refsum S Expression of EZH2 and Ki-67 in colorectal cancer and associations with treatment response and prognosis. Br J Cancer (2009) 101(8):1282–9. 10.1038/sj.bjc.6605333 19773751 PMC2768450

[18] Toll AD Dasgupta A Potoczek M Yeo CJ Kleer CG Brody JR Implications of enhancer of zeste homologue 2 expression in pancreatic ductal adenocarcinoma. Hum Pathol (2010) 41(9):1205–9. 10.1016/j.humpath.2010.03.004 20573371

[19] Zhai R Tang F Gong J Zhang J Lei B Li B The relationship between the expression of USP22, BMI1, and EZH2 in hepatocellular carcinoma and their impacts on prognosis. Onco Targets Ther (2016) 9:6987–98. 10.2147/OTT.S110985 27920552 PMC5125798

[20] Borbone E Troncone G Ferraro A Jasencakova Z Stojic L Esposito F Enhancer of zeste homolog 2 overexpression has a role in the development of anaplastic thyroid carcinomas. J Clin Endocrinol Metab (2011) 96(4):1029–38. 10.1210/jc.2010-1784 21289264

[21] Aziza EA Shimaa AA Ragab AA. Prognostic value of twist-1, E-cadherin and EZH2 in prostate cancer: an immunohistochemical study. Turk Patoloji Derg (2017) 1:198–210. 10.5146/tjpath.2016.01392 28832071

[22] Raman JD Mongan NP Tickoo SK Boorjian SA Scherr DS Gudas LJ. Increased expression of the polycomb group gene, EZH2, in transitional cell carcinoma of the bladder. Clin Cancer Res (2005) 11(24):8570–6. 10.1158/1078-0432.CCR-05-1047 16361539

[23] Jin M Yang Z Ye W Yu X Hua X. Prognostic significance of histone methyltransferase enhancer of zeste homolog 2 in patients with cervical squamous cell carcinoma. Oncol Lett (2015) 10(2):857–62. 10.3892/ol.2015.3319 26622583 PMC4508988

[24] Makk E Bálint L Cifra J Tornóczky T Oszter A Tóth A Robust expression of EZH2 in endocervical neoplastic lesions. Virchows Arch (2019) 475(1):95–104. 10.1007/s00428-019-02550-8 30903272 PMC6611890

[25] Zhou J Roh JW Bandyopadhyay S Chen Z Munkarah AR Hussein Y Overexpression of enhancer of zeste homolog 2 (EZH2) and focal adhesion kinase (FAK) in high grade endometrial carcinoma. Gynecol Oncol (2013) 128(2):344–8. 10.1016/j.ygyno.2012.07.128 22871469

[26] Krill L Deng W Eskander R Mutch D Zweizig S Hoang B Overexpression of enhance of zeste homolog 2 (EZH2) in endometrial carcinoma: an NRG oncology/gynecologic oncology group study. Gynecol Oncol (2020) 156(2):423–9. 10.1016/J.YGYNO.2019.12.003 31843273 PMC7103063

[27] Xu Y Li X Wang H Xie P Yan X Bai Y Hypermethylation of CDH13, DKK3 and FOXL2 promoters and the expression of EZH2 in ovary granulosa cell tumors. Mol Med Rep (2016) 14(3):2739–45. 10.3892/mmr.2016.5521 27431680

[28] Li H Cai Q Godwin AK Zhang R. Enhancer of zeste homolog 2 promotes the proliferation and invasion of epithelial ovarian cancer cells. Mol Cancer Res (2010) 8(12):1610–8. 10.1158/1541-7786.MCR-10-0398 21115743 PMC3059727

[29] Ramaglia M D’Angelo V Iannotta A Di Pinto D Pota E Affinita MC High EZH2 expression is correlated to metastatic disease in pediatric soft tissue sarcomas. Cancer Cell Int (2016) 16(1):59. 10.1186/S12935-016-0338-X 27471434 PMC4964052

[30] Adesso L Leoncini PP Dall’agnese A Walters ZS Verginelli F Boldrini R The Polycomb group (PcG) protein EZH2 supports the survival of PAX3-FOXO1 alveolar rhabdomyosarcoma by repressing FBXO32 (Atrogin1/MAFbx). Oncogene (2014) 33:4173–84. 10.1038/onc.2013.471 24213577

[31] Zhang N Zeng Z Li S Wang F Huang P. High expression of EZH2 as a marker for the differential diagnosis of malignant and benign myogenic tumors. Sci Rep (2018) 8:12331. 10.1038/s41598-018-30648-7 30120321 PMC6098067

[32] Shen JK Cote GM Gao Y Choy E Mankin HJ Hornicek FJ Targeting EZH2-mediated methylation of H3K27 inhibits proliferation and migration of Synovial Sarcoma *in vitro* . Sci Rep (2016) 6:25239. 10.1038/srep25239 27125524 PMC4850444

[33] Sun R Shen J Gao Y Zhou Y Yu Z Hornicek F Overexpression of EZH2 is associated with the poor prognosis in osteosarcoma and function analysis indicates a therapeutic potential. Oncotarget (2016) 7(25):38333–46. 10.18632/oncotarget.9518 27223261 PMC5122393

[34] Girard N Bazille C Lhuissier E Benateau H Llombart-Bosch A Boumediene K 3-Deazaneplanocin A (DZNep), an inhibitor of the histone methyltransferase EZH2, induces apoptosis and reduces cell migration in chondrosarcoma cells. PLoS One (2014) 9(5):e98176. 10.1371/JOURNAL.PONE.0098176 24852755 PMC4031152

[35] Cao Q Yu J Dhanasekaran SM Kim JH Mani RS Tomlins SA Repression of E-cadherin by the polycomb group protein EZH2 in cancer. Oncogene (2008) 27(58):7274–84. 10.1038/onc.2008.333 18806826 PMC2690514

[36] Ihira K Dong P Xiong Y Watari H Konno Y Hanley SJB EZH2 inhibition suppresses endometrial cancer progression via miR-361/Twist axis. Oncotarget (2017) 8(8):13509–20. 10.18632/oncotarget.14586 28088786 PMC5355116

[37] Ang PP Tan GC Karim N Wong YP. Diagnostic value of the EZH2 immunomarker in malignant effusion cytology. Acta Cytol (2020) 64(3):248–55. 10.1159/000501406 31352449

[38] Huvila J Pors J Thompson EF Gilks CB. Endometrial carcinoma: molecular subtypes, precursors and the role of pathology in early diagnosis. J Pathol (2021) 253(4):355–65. 10.1002/PATH.5608 33368243

[39] Buza N Tavassoli FA. Comparative analysis of P16 and P53 expression in uterine malignant mixed mullerian tumors. Int J Gynecol Pathol (2009) 28(6):514–21. 10.1097/PGP.0b013e3181a934e9 19851197

[40] WHO Classification of Tumours Editorial Board. Female genital tumours. 5th ed. International Agency for Research on Cancer (2020). Volume 4. Available at: https://publications.iarc.fr/Book-And-Report-Series/Who-Classification-Of-Tumours/Female-Genital-Tumours-2020 .

[41] Bosari S Lee AKC Viale G Heatley GJ Coggi G. Abnormal p53 immunoreactivity and prognosis in node-negative breast carcinomas with long-term follow-up. Virchows Arch A Pathol Anat Histopathol (1992) 421(4):291–5. 10.1007/BF01660975 1413493

[42] Bachmann IM Halvorsen OJ Collett K Stefansson IM Straume O Haukaas SA EZH2 expression is associated with high proliferation rate and aggressive tumor subgroups in cutaneous melanoma and cancers of the endometrium, prostate, and breast. J Clin Oncol (2006) 24:268–73. 10.1200/JCO.2005.01.5180 16330673

[43] Wagener N Macher-Goeppinger S Pritsch M Hüsing J Hoppe-Seyler K Schirmacher P Enhancer of zeste homolog 2 (EZH2) expression is an independent prognostic factor in renal cell carcinoma. BMC Cancer (2010) 10(1):524. 10.1186/1471-2407-10-524 20920340 PMC2958940

[44] Sasaki M Matsubara T Kakuda Y Sato Y Nakanuma Y. Immunostaining for polycomb group protein EZH2 and senescent marker p16INK4a may be useful to differentiate cholangiolocellular carcinoma from ductular reaction and bile duct adenoma. Am J Surg Pathol (2014) 38(3):364–9. 10.1097/PAS.0000000000000125 24487593

[45] Salvesen HB Iversen OE Akslen LA. Prognostic significance of angiogenesis and ki-67, p53, and p21 expression: a population-based endometrial carcinoma study. J Clin Oncol (1999) 17(5):1382–90. 10.1200/JCO.1999.17.5.1382 10334522

[46] Chen X Arend R Hamele-Bena D Tergas AI Hawver M Tong GX Uterine carcinosarcomas: clinical, histopathologic and immunohistochemical characteristics. Int J Gynecol Pathol (2017) 36(5):412–9. 10.1097/PGP.0000000000000346 28700424

[47] Köbel M Ronnett BM Singh N Soslow RA Gilks CB McCluggage WG. Interpretation of P53 immunohistochemistry in endometrial carcinomas: toward increased reproducibility. Int J Gynecol Pathol (2019) 38(1):S123–S131. 10.1097/PGP.0000000000000488 29517499 PMC6127005

[48] Rabban JT Garg K Ladwig NR Zaloudek CJ Devine WP. Cytoplasmic pattern p53 immunoexpression in pelvic and endometrial carcinomas with TP53 mutation involving nuclear localization domains: an uncommon but potential diagnostic pitfall with clinical implications. Am J Surg Pathol (2021) 45(11):1441–51. 10.1097/PAS.0000000000001713 33899789

[49] Schoonjans F Zalata A Depuydt CE Comhaire FH. MedCalc: a new computer program for medical statistics. Comput Methods Programs Biomed (1995) 48(3):257–62. 10.1016/0169-2607(95)01703-8 8925653

[50] Cohen J. A coefficient of agreement for nominal scales. Educ Psychol Meas (1960) 20(1):37–46. 10.1177/001316446002000104

[51] Engelsen IB Stefansson I Akslen LA Salvesen HB. Pathologic expression of p53 or p16 in preoperative curettage specimens identifies high-risk endometrial carcinomas. Am J Obstet Gynecol (2006) 195(4):979–86. 10.1016/j.ajog.2006.02.045 16677592

[52] Priya A Chaurasia JK P K Panwar H Yadav SK Kapoor N. Evaluation of immunohistochemical expression of enhancer of zeste homolog 2 (EZH2) and its association with clinicopathological variables in carcinoma cervix. Cureus (2023) 15(3):e36946. 10.7759/CUREUS.36946 37131568 PMC10148987

[53] Oki S Sone K Oda K Hamamoto R Ikemura M Maeda D Oncogenic histone methyltransferase EZH2: a novel prognostic marker with therapeutic potential in endometrial cancer. Oncotarget (2017) 8:40402–11. 10.18632/oncotarget.16316 28418882 PMC5522273

[54] Rao ZY Cai MY Yang GF He LR Mai SJ Hua WF EZH2 supports ovarian carcinoma cell invasion and/or metastasis via regulation of TGF-beta1 and is a predictor of outcome in ovarian carcinoma patients. Carcinogenesis (2010) 31(9):1576–83. 10.1093/carcin/bgq150 20668008

[55] Ding M Zhang H Li Z Wang C Chen J Shi L The polycomb group protein enhancer of zeste 2 is a novel therapeutic target for cervical cancer. Clin Exp Pharmacol Physiol (2015) 42(5):458–64. 10.1111/1440-1681.12382 25739318

[56] Bitler BG Aird KM Garipov A Li H Amatangelo M Kossenkov AV Synthetic lethality by targeting EZH2 methyltransferase activity in ARID1A-mutated cancers. Nat Med (2015) 21(3):231–8. 10.1038/NM.3799 25686104 PMC4352133

[57] Roh JW Eun Choi J Dong Han H Hu W Matsuo K Nishimura M Clinical and biological significance of EZH2 expression in endometrial cancer. Cancer Biol Ther (2019) 21:147–56. 10.1080/15384047.2019.1672455 31640461 PMC7012102

[58] Shi L Zhang Q Zhu S Tang Q Chen X Lan R Pharmacological inhibition of EZH2 using a covalent inhibitor suppresses human ovarian cancer cell migration and invasion. Mol Cell Biochem (2023). 10.1007/S11010-023-04767-3 37199893

[59] Abargel A Avinoach I Kravtsov V Boaz M Glezerman M Menczer J. Expression of p27 and p53: comparative analysis of uterine carcinosarcoma and endometrial carcinoma. Int J Gynecol Cancer (2004) 14(2):354–9. 10.1111/j.1048-891x.2004.014221.x 15086737

[60] Menczer J Kravtsov V Levy T Berger E Glezerman M Avinoach I. Expression of c-kit in uterine carcinosarcoma. Gynecol Oncol (2005) 96(1):210–5. 10.1016/j.ygyno.2004.09.045 15589603

[61] Menczer J Schreiber L Sukmanov O Kravtsov V Berger E Golan A COX-2 expression in uterine carcinosarcoma. Acta Obstet Gynecol Scand (2010) 89(1):120–5. 10.3109/00016340903342006 19900134

[62] Livasy CA Reading FC Moore DT Boggess JF Lininger RA. EGFR expression and HER2/neu overexpression/amplification in endometrial carcinosarcoma. Gynecol Oncol (2006) 100(1):101–6. 10.1016/j.ygyno.2005.07.124 16157366

[63] Saglam O Husain S Toruner G. AKT, EGFR, C-ErbB-2, and C-kit expression in uterine carcinosarcoma. Int J Gynecol Pathol (2013) 32(5):493–500. 10.1097/PGP.0b013e31827fedef 23896716

[64] Rottmann D Snir OL Wu X Wong S Hui P Santin AD HER2 testing of gynecologic carcinosarcomas: tumor stratification for potential targeted therapy. Mod Pathol (2020) 33(1):118–27. 10.1038/S41379-019-0358-x 31477811

[65] Jenkins TM Cantrell LA Stoler MH Mills AM. HER2 overexpression and amplification in uterine carcinosarcomas with serous morphology. Am J Surg Pathol (2022) 46(4):435–42. 10.1097/PAS.0000000000001870 35125452

[66] Murali R Davidson B Fadare O Carlson JA Crum CP Gilks CB High-grade endometrial carcinomas: morphologic and immunohistochemical features, diagnostic challenges and recommendations. Int J Gynecol Pathol (2019) 38(1):S40–S63. 10.1097/PGP.0000000000000491 30550483 PMC6296248

[67] Yemelyanova A Ji H Shih IM Wang TL Wu LSF Ronnett BM. Utility of p16 expression for distinction of uterine serous carcinomas from endometrial endometrioid and endocervical adenocarcinomas: immunohistochemical analysis of 201 cases. Am J Surg Pathol (2009) 33(10):1504–14. 10.1097/PAS.0B013E3181AC35F5 19623034

[68] Gilks CB Oliva E Soslow RA. Poor interobserver reproducibility in the diagnosis of high-grade endometrial carcinoma. Am J Surg Pathol (2013) 37(6):874–81. 10.1097/PAS.0B013E31827F576A 23629444

[69] Garg K Leitao MM Wynveen CA Sica GL Shia J Shi W p53 overexpression in morphologically ambiguous endometrial carcinomas correlates with adverse clinical outcomes. Mod Pathol (2010) 23(1):80–92. 10.1038/MODPATHOL.2009.153 19855378

[70] Tashiro H Isacson C Levine R Kurman RJ Cho KR Hedrick L. p53 gene mutations are common in uterine serous carcinoma and occur early in their pathogenesis. Am J Pathol (1997) 150(1):177–85. Available from: https://www.ncbi.nlm.nih.gov/pmc/articles/PMC1858541/ (Accessed July 27, 2023). 9006334 PMC1858541

[71] Soslow RA Shen PUF Chung MH Isacson C. Distinctive p53 and mdm2 immunohistochemical expression profiles suggest different pathogenetic pathways in poorly differentiated endometrial carcinoma. Int J Gynecol Pathol (1998) 17(2):129–34. 10.1097/00004347-199804000-00006 9553809

[72] Gu Y Zhang J Guan H. Expression of EZH2 in endometrial carcinoma and its effects on proliferation and invasion of endometrial carcinoma cells. Oncol Lett (2017) 14(6):7191–6. 10.3892/ol.2017.7171 29344151 PMC5754892

[73] Castilla MÁ Moreno-Bueno G Romero-Pérez L Van De Vijver K Biscuola M López-García MÁ Micro-RNA signature of the epithelial-mesenchymal transition in endometrial carcinosarcoma. J Pathol (2011) 223(1):72–80. 10.1002/PATH.2802 21125666

[74] Chiyoda T Tsuda H Tanaka H Kataoka F Nomura H Nishimura S Expression profiles of carcinosarcoma of the uterine corpus—are these similar to carcinoma or sarcoma? Genes, Chromosom Cancer (2012) 51(3):229–39. 10.1002/GCC.20947 22072501

[75] McCluggage WG. Uterine carcinosarcomas (malignant mixed Mullerian tumors) are metaplastic carcinomas. Int J Gynecol Cancer (2002) 12(6):687–90. 10.1046/j.1525-1438.2002.01151.x 12445244

[76] Kanthan R Senger JLB Diudea D. Malignant mixed Mullerian tumors of the uterus: histopathological evaluation of cell cycle and apoptotic regulatory proteins. World J Surg Oncol (2010) 8:60. 10.1186/1477-7819-8-60 20642852 PMC2913917

[77] Köbel M Kang EY. The many uses of p53 immunohistochemistry in gynecological pathology: proceedings of the ISGyP companion society session at the 2020 USCAP annual9 meeting. Int J Gynecol Pathol (2021) 40(1):32–40. 10.1097/PGP.0000000000000725 33290354

[78] Rabban JT Blake Gilks C Malpica A Matias-Guiu X Mittal K Mutter GL Issues in the differential diagnosis of uterine low-grade endometrioid carcinoma, including mixed endometrial carcinomas: recommendations from the international society of gynecological pathologists. Int J Gynecol Pathol (2019) 38(1):S25–S39. 10.1097/PGP.0000000000000512 30550482 PMC6296831

[79] Kunc M Gabrych A Rekawiecki B Gorczyński A Haybaeck J Biernat W Immunohistochemical evaluation of mismatch repair proteins and p53 expression in extrauterine carcinosarcoma/sarcomatoid carcinoma. Wspolczesna Onkol (2020) 24(1):1–4. 10.5114/WO.2020.94718 PMC7265955 32514231

[80] Liu FS Kohler MF Marks JR Bast RC Jr Boyd J Berchuck A. Mutation and overexpression of the P53 tumor suppressor gene frequently occurs in uterine and ovarian sarcomas. Obstet Gynecol (1994) 83(1):118–24. 8272291

[81] De Jong RA Nijman HW Wijbrandi TF Reyners AK Boezen HM Hollema H. Molecular markers and clinical behavior of uterine carcinosarcomas: focus on the epithelial tumor component. Mod Pathol (2011) 24(10):1368–79. 10.1038/MODPATHOL.2011.88 21572397

[82] Köbel M Piskorz AM Lee S Lui S LePage C Marass F Optimized p53 immunohistochemistry is an accurate predictor of TP53 mutation in ovarian carcinoma. J Pathol Clin Res (2016) 2(4):247–58. 10.1002/CJP2.53 27840695 PMC5091634

[83] da Costa LT Dos Anjos LG Kagohara LT Torrezan GT De Paula CAA Baracat EC The mutational repertoire of uterine sarcomas and carcinosarcomas in a Brazilian cohort: a preliminary study. Clinics (2021) 76:e2324–15. 10.6061/CLINICS/2021/E2324 33503190 PMC7798418

[84] Zhao S Bellone S Lopez S Thakral D Schwab C English DP Mutational landscape of uterine and ovarian carcinosarcomas implicates histone genes in epithelial-mesenchymal transition. Proc Natl Acad Sci U S A (2016) 113(43):12238–43. 10.1073/pnas.1614120113 27791010 PMC5087050

[85] Taylor NP Zighelboim I Huettner PC Powell MA Gibb RK Rader JS DNA mismatch repair and TP53 defects are early events in uterine carcinosarcoma tumorigenesis. Mod Pathol (2006) 19:1333–8. 10.1038/modpathol.3800654 16810312

[86] Jones S Stransky N McCord CL Cerami E Lagowski J Kelly D Genomic analyses of gynaecologic carcinosarcomas reveal frequent mutations in chromatin remodelling genes. Nat Commun (2014) 5:5006. 10.1038/NCOMMS6006 25233892 PMC4354107

[87] McConechy MK Ding J Cheang MCU Wiegand K Senz J Tone A Use of mutation profiles to refine the classification of endometrialcarcinomas. J Pathol (2012) 228(1):20–30. 10.1002/PATH.4056 22653804 PMC3939694

[88] Di Fiore R Suleiman S Drago-Ferrante R Subbannayya Y Vasileva-Slaveva M Yordanov A The role of FBXW7 in gynecologic malignancies. Cells (2023) 12(10):1415. 10.3390/cells12101415 37408248 PMC10216672

[89] Cuevas IC Sahoo SS Kumar A Zhang H Westcott J Aguilar M Fbxw7 is a driver of uterine carcinosarcoma by promoting epithelial-mesenchymal transition. Proc Natl Acad Sci U S A (2019) 116(51):25880–90. 10.1073/pnas.1911310116 31772025 PMC6926017

